# *SDHA* secondary findings in germline testing: counseling and surveillance considerations

**DOI:** 10.1530/EO-23-0043

**Published:** 2024-05-13

**Authors:** Catherine M Skefos, Pamela L Brock, Erica Blouch, Samantha E Greenberg

**Affiliations:** 1 The University of Texas MD Anderson Cancer Center, Clinical Cancer Genetics Program, Houston, Texas, USA; 2 The Ohio State University College of Medicine, Division of Human Genetics, Comprehensive Cancer Center, Columbus, Ohio, USA; 3 Massachusetts General Hospital Cancer Center, Boston, Massachusetts, USA; 4 Department of Health Care Sciences, UT Southwestern Medical Center, Dallas, Texas, USA

**Keywords:** genetic counseling, hereditary paraganglioma, incidental findings, paraganglioma, SDHx, secondary findings

## Abstract

This commentary explores the complexities faced by clinicians when encountering a secondary *SDHA* pathogenic variant (PV) in patients without a personal or family history of *SDHA*-related tumors. The increasing use of germline multi-gene panel testing has led to a rise in such secondary findings, necessitating a nuanced approach to counseling, surveillance, and decision-making. We aim to discuss the current data surrounding the penetrance of *SDHA* PVs, the spectrum of screening guidelines, recommendations for educating individuals and families about their secondary findings, and the need for future research to optimize care for these individuals. Practical recommendations for clinicians dealing with patients with secondary* SDHA* findings include acknowledging the limitations of existing guidelines, fostering shared decision-making, and considering specialist referrals. Overall, the evolving landscape of *SDHA* penetrance data warrants ongoing reassessment of surveillance approaches.

## Introduction

A 44-year-old woman is diagnosed with breast cancer and undergoes germline multi-gene panel testing (MGPT). The results reveal no findings related to her breast cancer diagnosis, but a pathogenic variant (PV) in the *SDHA* gene. She has no personal or family history of *SDHA-*related tumors. The patient has four children ranging from age 8 to 20, who have not had genetic testing. After disclosure of the genetic testing results, this patient has many questions:

- What is my chance of developing an *SDHA*-related tumor?
- What screening is recommended for me?
- What are the next steps for my children?
- How do I juggle this new finding with my current healthcare needs?

For the clinician disclosing these results and evaluating this patient, the answers to these questions are complicated and continue to evolve based on current data and expert/consensus guidelines. This commentary aims to inform a clinician’s approach to a secondary *SDHA* finding by reviewing the current state of *SDHA* penetrance data, screening recommendations, and future research needs.

The advent of next-generation sequencing and the subsequent decrease in germline testing costs has led to an increase in MGPT for patients with a personal and family history of cancer (Schienda & Stopfer 2020). As more genes are tested, the chances of discovering an unexpected, or secondary, finding increase (Zhang *et al.* 2015, Mandelker *et al.* 2017). The National Society of Genetic Counselors defines a secondary finding as a finding in a gene that is purposely analyzed as part of the test but unrelated to the primary indication for testing (National Society of Genetic Counselors 2023). This contrasts with an incidental finding, such as a discrepancy in sex chromosome composition detected during quality control, which is unrelated to the initial testing purpose. Though ‘secondary’ and ‘incidental’ are often used interchangeably, the term secondary finding will be used to describe an *SDHA* PV identified in a patient with no personal or family history of *SDHA*-related tumors.

## *SDHA-*related hereditary paraganglioma–pheochromocytoma syndrome

The *SDHA* gene encodes one of four subunits in the succinate dehydrogenase (SDH) complex, which functions as tumor suppressors. Germline *SDHA* PVs are associated with an increased risk of paraganglioma (PGL), pheochromocytoma (PCC), gastrointestinal stromal tumor (GIST), and renal cell carcinoma (van der Tuin *et al.* 2018). This hereditary predisposition falls under the spectrum of hereditary paraganglioma–pheochromocytoma (PPGL) syndromes. Additional genes that cause hereditary PPGL include the other SDHx genes (*SDHB*,* SDHC*,* SDHD*, and *SDHAF2*), *MAX*, and *TMEM127*. PVs in *RET, FH, VHL, NF1*, and other genes are also known to increase the risk of PGL and/or PCC.

Although individuals with hereditary PPGL syndrome may develop any SDHx*-*related tumors, the penetrance and phenotype of hereditary PPGL syndrome differ significantly depending on the gene. PVs in *MAX* and *TMEM127* most often cause PCC, whereas head/neck PGL are more common in patients with PVs in *SDHC,* *SDHD*, and *SDHAF2*. *SDHB* and *SDHA* PVs have been associated with PPGL across multiple locations, including abdominopelvic and head/neck PGLs. Pathogenic variants in each gene confer a specific risk for developing a related tumor (penetrance), with the highest risk associated with paternally inherited *SDHD* PVs (van der Tuin *et al.* 2018).

Approximately 4% of individuals with hereditary PPGL syndrome have an *SDHA* PV. A retrospective cohort study of 393 patients with prior negative genetic testing of *SDHB*,* SDHC*,* SDHD*,and *SDHAF2* identified 30 patients with an *SDHA* PV. The most common presentation was a single sympathetic PGL, followed by head/neck PGL, and PCC (van der Tuin *et al.* 2018). An additional series of 34 individuals, both probands and their family members, with an *SDHA* PV showed that head/neck PGL was the most common manifestation in 44% of affected patients. Four patients in this series also had metastatic disease (Bausch *et al.* 2017). In both studies, a vast majority of patients lacked a family history of *SDHA*-related tumors. The earliest reported tumor diagnosis in these studies occurred at age 8 and 15, respectively, but most patients were not diagnosed until their fifth decade of life.

Additional attention has recently been paid to the metastatic potential of *SDHA*-related PPGL, and it is believed that these PPGLs have a higher propensity for spread than initially reported. A series of 10 patients with metastatic PPGL associated with an *SDHA* PV showed a similar disease course to *SDHB*-related tumors (Jha *et al.* 2019); additional studies have also identified a proportion of metastatic disease ranging from 10% to 33% (Bausch *et al.* 2017, Tufton *et al.* 2017, van der Tuin *et al.* 2018).

Both GIST and RCC have been reported for individuals with an *SDHA* PV. Among patients with SDH-deficient GISTs, an estimated 30% have a germline *SDHA* PV (Schipani *et al.* 2023). *SDHB*, *SDHC*, and *SDHD* PVs have also been reported in SDH-deficient GISTs, prompting the recommendation for germline testing of all four genes in individuals with wild-type GIST. SDH-deficient RCC is likewise a relatively rare subset of kidney cancers but has been associated with germline PVs in both *SDHA* and *SDHB* (McEvoy *et al.* 2018).

### Penetrance of *SDHA* pathogenic variants

Optimizing *SDHA* surveillance options for patients requires consideration of penetrance and evaluation of the threshold at which screening is optimally beneficial for early tumor detection (Pinsky 2015). The estimated lifetime tumor risk for individuals with an *SDHA* pathogenic variant continues to be revised as new data is disseminated. Penetrance estimates for individuals with *SDHA* PVs range from 0.1% (Maniam *et al.* 2018) to 10% (van der Tuin *et al.* 2018). As additional cases are identified with secondary *SDHA* PVs, this number is expected to evolve.

Multiple studies have attempted to evaluate penetrance through literature reviews and/or institutional cohorts. Benn *et al.* (2018) used a Bayesian approach to evaluate unrelated Australian and UK subjects with paragangliomas, estimating lifetime disease penetrance to be 1.7% in those with *SDHA* PVs. In contrast, a multi-center cohort study of patients with PPGL in the Netherlands calculated 10% penetrance by age 70, though the 95% CI ranged from 0% to 21% (van der Tuin *et al.* 2018). Additional studies utilize literature review, with or without institutional cohorts, to derive *SDHA* penetrance values. Comparing available *SDHA* cases in the literature to GnomAD generates a penetrance range from 0.1% to 4.9% (Maniam *et al.* 2018). These estimates demonstrate wide variability due to the differences in methodology and ascertainment between studies. These studies are summarized in Table 1.

**Table 1:** Summary of literature analyzing penetrance in individuals with *SDHA* PVs.

First author, year, and country	Title	Sample and ascertainment	Study design	Penetrance
Benn *et al.* (2018) Australia, UK	Bayesian approach to determine penetrance of pathogenic SDH variants.	575 Australian and 1275 UK individuals with PPGL and genetic testing.	Comparing allelic frequencies in cases vs controls from ExAC.	1.7%
Casey *et al.* (2017) UK	SDHA related tumorigenesis: a new case series and literature review for variant interpretation and pathogenicity.	Literature review: 47 individuals with PPGL and *SDHA* variant UK cohort: 15 PPGL cases and *SDHA* variant.		Found in healthy individuals at frequency between 1/1000 and 1/10,000
Maniam *et al.* (2018) N/A	Pathogenicity and penetrance of germline *SDHA* variants in pheochromocytoma and paraganglioma (PPGL).	PubMed: PPGL with heterozygous germline *SDHA* variants. GnomAD: All reported *SDHA* variants in GnomAD.	Comparison of *SDHA* variant frequency in PPGL literature and GnomAD cohorts.	0.1–4.9%
Van der Tuin *et al.* (2018) Netherlands	Clinical aspects of *SDHA*-related pheochromocytoma and paraganglioma: A nationwide study.	393 individuals with PPGL; 30 variant carriers and 56 non-index carriers.	Nationwide retrospective cohort study.	10% at age 70 years

ExAC, Exome Aggregation Consortium; GnomAD, Genome Aggregation Database; PPGL, pheochromocytoma and paraganglioma.

The penetrance of *SDHA* PVs appears to be significantly lower than PVs in *SDHB* and *SDHD*, the most studied of the SDHx genes*,* whose penetrance ranges from 22% to 43% in non-probands (Andrews *et al.* 2018, Benn *et al.* 2018). In the same studies, *SDHC* penetrance ranges from 8% to 25% in non-probands, which overlaps with both *SDHA* and *SDHB/D.* More research is necessary to understand the penetrance of *SDHC* PVs. Therefore*,* whether the same SDHx surveillance guidelines should be applied across all SDHx genes is unknown. Although currently, most guidelines include all SDHx genes in their recommendations, recent publications suggest modifying the surveillance approach for individuals with an *SDHA* PV (Hanson *et al.* 2023).

### *SDHA*PV as a secondary finding

Recent years have demonstrated a rise in reports of *SDHA* PVs as a secondary finding. A 5-year retrospective review of hereditary PPGL at a single institution identified 83 patients with a PV in an SDHx gene or in *TMEM127*, 19 (23%) of which were secondary findings. Of the secondary findings, at least 30% were in *SDHA* (personal communication, Mersch 2023). A similar study identified 22 patients (12 *SDHA)* with SDHx secondary findings over the past 5 years (Buchanan *et al.* 2022). Regarding the *SDHA* gene specifically, a recent abstract described 37 patients with an *SDHA* PV, 33 (89%) of which were secondary findings (Kohlmann *et al.* 2023). One study from an academic cancer center investigated the frequency of germline *SDHA* PV (*n* = 10) in a cohort of ~5000 patients with solid tumors (Dubard Gault *et al.* 2018). Two of the 10 patients had a GIST diagnosis, which falls within the *SDHA* tumor spectrum. The remaining eight patients had a variety of tumors not typically associated with an *SDHA* mutation. Follow-up testing revealed a somatic second hit in a GIST and a neuroblastoma, indicating an oncogenic role for *SDHA* in these tumors. Somatic findings were absent in the tumors not related to the *SDHA* phenotype.

### Surveillance guidelines

Table 2 summarizes existing surveillance guidelines relevant to individuals with *SDHA* PVs. Guidelines are largely written by and for providers within a specific country (USA, UK, Sweden, or Australia/New Zealand). One publication represents the main source of international guidance (Amar *et al.* 2021). Most guidelines broadly address hereditary PPGL surveillance, while the UK guideline is written specifically for individuals with *SDHA* PV (Hanson *et al.* 2023). This UK guideline is also unique in that it recommends neither surveillance in individuals with *SDHA* found as a secondary finding nor cascade testing for their family members.

**Table 2:** Summary of current *SDHx*-related surveillance recommendations.

Guideline author/group	Origin/contributors	Age to start BP/biochemical surveillance (interval)	Age to start imaging (interval) and special recommendations (if not recommending MRI)	Comments/distinguishing features
AACR Childhood Cancer Predisposition Workshop Rednam *et al.* (2017)	International cohort of leading pediatric cancer experts from the USA and Canada.	6–8 years (annual).	6–8 years (every 2 years).	The guideline is targeted for the pediatric population, so recommendations may not apply to adults. Biochemical assessment also includes plasma methoxytyramine.
Amar *et al.* (2021)	International panel of 29 PPGL experts from 12 countries. Delphi method for consensus building.	10–15 years, (biennial in children and annual in adults).	10–15 years (every 2–3 years) PET-CT for initial surveillance in adults.	Analyzed each gene separately but resulted in similar recommendations for all the SDHx genes, except regarding the age to start surveillance.
eviQ SDHA, SDHB, or SDHC-related risk management (version 3) www.eviq.org.au	Consensus guideline from committee of genetic counselors, geneticists, and cancer genetics researchers from Australia and New Zealand. Updated every 2 years.	18 years (annual).	18 years (every 5 years) 68Ga-Dotatate PET-CT/MRI once with initial imaging.	Age to start surveillance differs based on SDHx gene. SDHA-specific statement: SDHA PV demonstrate low penetrance. Particular care should be taken in formulating follow-up plans for asymptomatic individuals with SDHA pathogenic variants to avoid over-surveillance.
Hanson *et al.* (2023) on behalf of UK Cancer Genetics Centres	Survey of 24 regional genetics centers in UK.	10, only if positive family history (annual).	15, only if positive family history (every 3–5 years).	Only SDHA-specific guideline. Cascade testing and surveillance only recommended for probands (or FDR of probands) with an associated tumor.
Muth *et al.* (2019)	Panel of nine PPGL experts from Sweden.	5 years before age of diagnosis in family (annual).	5 years before age of diagnosis in family (every 2 years, increase to every 3 if normal).	Does not address the scenario of incidental SDHA but only recommend ongoing surveillance in FDR and one time assessment in SDR.
National Comprehensive Cancer Network (NCCN) Version 1.2023	Consensus guideline from expert panel with representation from 33 US-based cancer centers. Updated annually.	10–15 years (annual).	10–15 years (every 2–3 years).	SDHA-specific statement: Consideration can be given to modified surveillance intervals given low penetrance.

AACR, American Association for Cancer Research; FDR, first-degree relative; PPGL, pheochromocytoma and paraganglioma; PV, pathogenic variant; SDR, second-degree relative.

#### Clinical surveillance

All guidelines recommend annual blood pressure monitoring and clinical assessment, typically starting at the age of biochemical surveillance. Amar *et al.* (2021) recommend a questionnaire to aid in the assessment of clinical symptoms.

#### Biochemical assessment

Most guidelines recommend biochemical analysis (such as plasma metanephrines) annually, but international guidelines recommend pediatric biennial surveillance and annual surveillance in adults. The guidelines vary in the preference of metanephrine evaluation via plasma or urine.

#### Imaging

All guidelines recommend full-body imaging, with most recommending MRI (preferably full-body or skull base to pelvis) as the preferred imaging modality. However, multiple guidelines suggest the consideration of PET/CT and ^68^Ga-Dotatate imaging options.

#### Surveillance cessation

Recommendations vary, with multiple suggesting that after age 70, surveillance tests should be prolonged to every 5 years and follow-up should be stopped at age 80 (Amar *et al.* 2021).

It should be acknowledged that the guidelines for hereditary PPGL surveillance above require regular clinical follow-up, with specialized testing protocols, and are largely written by individuals who work at large, well-resourced, academic centers. Additionally, the authors of this commentary likewise work in such centers and acknowledge that patients and providers outside of these settings will face greater barriers to accessing specialty care. Regardless of expertise, the discrepancies in the guidelines raise challenges for any provider to determine appropriate surveillance as secondary *SDHA* PVs become commonplace. In other hereditary cancer syndromes, there is a shift from broad condition surveillance guidelines to gene-specific recommendations. It is possible that *SDHA* is in the middle of this shift, and guidelines will be standardized soon. In the meantime, multiple considerations should be incorporated to identify the best approach for the individual and their family given resources available and patient-specific concerns.

### Counseling a patient with a secondary *SDHA* finding

It is imperative to engage in shared decision-making regarding surveillance and cascade testing when providing care to someone with a secondary *SDHA* PV. Providers unaccustomed to managing patients with hereditary PPGL syndromes should consider referral to a physician/center familiar with this indication after initial discussion with the patient. Several resources exist to identify experts in the field. Examples include utilizing a provider search through the Pheo-Para Alliance (pheopara.org) or Endocrine Society’s Find an Endocrinologist feature (https://www.endocrine.org/patient-engagement/find-an-endocrinologist-directory – search term ‘Endocrine Cancer and Neoplasia’).

Despite recent efforts to standardize gene-specific recommendations, discrepancies between guidelines can be challenging for providers to navigate. Hanson *et al.* (2023) is the only guideline that suggests no surveillance or predictive testing in families with secondary *SDHA* finding. Hanson *et al.* also suggest SDHA/SDHB IHC analysis in tumors not associated with *SDHA* PV (such as breast cancer) to determine if probands should be included in surveillance recommendations, despite a lack of data to suggest that IHC analysis is effective across non-SDHx tumor types. The recommendations by this group are likely influenced by sentiment in the cancer genetics community that, given the penetrance, patients with *SDHA*-associated hereditary PPGL are being over-screened. Only offering surveillance in patients/families with known *SDHA*-associated tumors falsely implies that data suggest the tumor risk is different based on family history, even though an absence of a family history of related tumors is not overly reassuring since the majority of probands with an SDHA-related tumor likewise have no associated family history. Limiting surveillance in this way could lead to inequalities in access or false reassurance for patients with limited family contact or information about their medical history.

In contrast, acknowledging and discussing the penetrance of *SDHA* PVs and the broad array of acceptable surveillance guidelines allows patients to take ownership in determining a surveillance plan that addresses their needs and concerns. Ultimately, patients and their families should understand that at this time there is no ‘one-size-fits-all’ surveillance recommendation for *SDHA-*related hereditary PPGL syndrome. As MGPT identifies an increasing volume of SDHA PV carriers, standardized screening based on evolving evidence will be required. Any surveillance plan put into action should be open to change as our understanding of the penetrance and clinical course of this condition evolves.

### Predictive testing

Apart from Hanson *et al.* (2023), a majority of guidelines recommend predictive testing for at-risk relatives in the setting of a secondary *SDHA* PV. Patients undergoing predictive testing should be aware of the benefits and limitations of the Genetic Information and Nondiscrimination Act. Discrepancies between guidelines also raise the question of whether to test minors for familial *SDHA* PVs. In contrast to high penetrance genes, no clear data exist to demonstrate the benefit of screening during childhood for *SDHA* PV carriers. Providers and families must balance the possible benefits of surveillance with the ethical tenant of autonomy, or whether the future adult should be allowed to make their own decisions about knowing their genetic status (Dondorp *et al.* 2021). Shared decision-making between providers and patients considering predictive testing for themselves or their children should include a discussion regarding the evolving *SDHA* penetrance data as well as the impact of rigorous surveillance on children and families.

### Key takeaways

For providers who see patients with an *SDHA* secondary finding as described in the opening vignette, we recommend the following takeaways:

- Acknowledge that existing guidelines addressing hereditary PPGL were written primarily in the context of *SDHB* and *SDHD* research.
- Consider discussing the following with the patient and family: reduced penetrance, variability between guidelines, range of acceptable surveillance recommendations, and symptom awareness.
- Encourage shared decision-making regarding surveillance and cascade testing.
- Highlight the evolving data and guidelines in order to provide anticipatory guidance that recommendations may change over time. Encourage patients to follow up with surveillance as mutually determined and reevaluate surveillance recommendations and decisions at subsequent appointments.
- If a patient chooses not to pursue surveillance, encourage them to re-engage if concerns arise and to check in periodically for updated recommendations.
- Consider referral to a physician/center familiar with hereditary PPGL.

As evidenced in this commentary, it is clear that current and future research will dictate shifts in these recommendations over time.

### Future directions

The growing rate at which *SDHA* PVs are identified in apparently unaffected individuals warrants additional research. Larger studies are needed to rule out possible associations of *SDHA* PVs with other cancer types beyond what is currently described. Furthermore, determining optimal surveillance guidelines requires clarifying if tumor risks differ for families with an *SDHA* PV based on family history. Subsequently, standardizing guidelines across specialties and countries ensures that individuals with the same *SDHA* findings receive similar care. Further research must seek to understand the patient perspective and resource needs in secondary cancer genetic findings. Although no studies have specifically looked at reactions to secondary findings in *SDHA*, data from secondary findings in exome/genome sequencing reveals that patients typically do not report long-term, negative impacts after being told of a secondary finding (Cheung *et al.* 2022). Whether response may differ when a secondary finding has low penetrance, like an *SDHA* mutation, is yet to be determined. Overall, current evidence regarding the low penetrance associated with *SDHA* PVs may not justify the same rigorous surveillance approach as in other hereditary PPGL syndromes. Consideration should be given to the development of gene-specific clinical management recommendations for individuals with hereditary PPGL.

## Declaration of interest

The authors declare that there is no conflict of interest that could be perceived as prejudicing the impartiality of this commentary.

## Funding

This work did not receive any specific grant from any funding agency in the public, commercial, or not-for-profit sector.

## Author contribution statement

All authors conceived the idea for the commentary and contributed to its development, including investigation of the topic, writing of the initial commentary, and contributing to its revision and review.
